# The Culture Dish Surface Influences the Phenotype and Cytokine Production of Human Monocyte-Derived Dendritic Cells

**DOI:** 10.3389/fimmu.2019.02352

**Published:** 2019-10-02

**Authors:** Alexander Sauter, Dag Heiro Yi, Yayan Li, Sabine Roersma, Silke Appel

**Affiliations:** ^1^Department of Biomedicine, University of Bergen, Bergen, Norway; ^2^Broegelmann Research Laboratory, Department of Clinical Science, University of Bergen, Bergen, Norway

**Keywords:** homotypic clusters, monocytes, monocyte-derived dendritic cells, immunogen, tolerogen, adhesion, non-adherent culture plate, cytokines

## Abstract

Monocyte-derived dendritic cells (moDC) are an important scientific and clinical source of functional dendritic cells (DC). However, the optimization of the generation process has to date mainly been limited to the variation of soluble factors. In this study, we investigated the impact of the cell culture dish surface on phenotype and cytokine profile. We compared a standard cell culture dish to a non-adherent culture dish for two immunogenic maturation conditions, two tolerogenic conditions, and an unstimulated control. Phenotype, cytokine profile and T cell stimulatory capacity were determined after a 3-day culture. Light microscopy revealed an increase in homotypic cluster formation correlated with the use of non-adherent surfaces, which could be reduced by using blocking antibodies against CD18. All surface markers analyzed showed moderate to strong differences depending on the culture dish surface, including significantly decreased expression of key maturation markers such as CD80, CD86, and CCR7 as well as PD-L1 on cells stimulated with the Jonuleit cytokine cocktail cultured on a non-adherent surface. Significant differences in the secretion of many cytokines were observed, especially for cells stimulated with LPS, with over 10-fold decreased secretion of IL-10, IL12-p40, and TNF-α from the cells cultured on the non-adherent surface. All immunogenic moDC populations showed similar capacity to induce antigen-specific T cells. These results provide evidence that the DC phenotype depends on the surface used during moDC generation. This has important implications for the optimization of DC-based immunotherapy development and underlines that the local surrounding can interfere with the final DC population beyond the soluble factors.

## Introduction

Dendritic cells (DC), positioned between the innate and adaptive immune system, play a central role in a great variety of immunological settings. They play an important role for the pathogenesis of many diseases, and are increasingly also under investigation as a clinical tool to treat a great diversity of different challenging conditions, ranging from cancer to autoimmunity (1). The role of DC in medicine has already been highlighted by their discoverer Ralph M. Steinman (1, 2), ranging from infectious diseases over autoimmunity to cancer (3–5). However, the complexity of the pathogenic settings in the immune system and the diversity of DC subtypes are contributing to an enduring challenge to understand and apply DC biology. Many obstacles have to be overcome on the way to clinics, but the understanding of the *in vitro* system for the development of DC applications is of special importance. As DC are a central sensing unit collecting all information before a possible activation of the adaptive immune system, it is not surprising that the culturing environment can have a great impact on the cellular phenotype and thus on the induced immune response. Commonly, blood monocytes are the major source of cells to generate DC *ex vivo*, mainly because they are readily accessible. Monocyte-derived DC (moDC) are then generated by use of conditioning soluble factors, usually a combination of GM-CSF and IL-4, to induce the DC program in monocytes, followed by a maturation cocktail that mimics an *in vivo* maturation condition leading to the desired phenotype. For example, one of the commonly used maturation cocktails for immunogenic DC is one that imitates an inflammatory situation in the skin (referred to as “Jonuleit cocktail”), containing IL-1β, IL-6, TNF, and prostaglandin E2 (PGE_2_) (6). Moreover, serum-free formulations are recommended to ensure reproducibility and achieve compliance with clinical requirements (7). However, only the impact of soluble factors is commonly considered, the adhesional culture properties are hugely ignored in most *in vitro* protocols. If mentioned at all, standard cell culture plates are recommended. Alone the *in vivo* regulation of DC adhesion upon maturation (8, 9) gives an indication that adhesional signaling might be of importance in a potentially more diverse way than can be expected from an unspecific surface of a plastic cell culture dish. In connection with the culturing conditions, we observed an early increase in DC markers on immature DC when cultured on non-adherent surfaces compared to standard cell culture dishes (10). In the same study, we observed an increase in homotypic clustering of the cells on non-adherent surfaces compared to cells on standard cell culture plates. Thus, the choice of the culture dish can potentially have a significant impact on the DC phenotype and function by either supporting the early, integrin-mediated adhesion followed by low homotypic clustering, or by reducing culture dish interactions leading to an increase in clustering and thus cluster-mediated cell-cell interactions.

The aim of the present study was to investigate the effect of the culture dish surface on the phenotype and the cytokine production of differentially stimulated immunogenic and tolerogenic moDC populations. We found that both phenotype and cytokine secretion are modulated in a treatment-dependent manner. Moreover, using blocking antibodies, we determined CD18 as the most important molecule for the homotypic cluster formation.

## Materials and Methods

### Dendritic Cell Generation

Freshly drawn peripheral blood was collected from 19 healthy volunteers into BD Vacutainer ACD-A 10 ml citrate tubes (BD, Franklin Lakes, USA). Informed consent was obtained from all donors. The study was approved by the regional ethical committee Western Norway (REK Vest; #2009/686). The age of the donors was ranging from 23 to 67 years. Monocytes were isolated as described previously (10). In short, peripheral blood mononuclear cells (PBMC) were isolated by density gradient centrifugation using Lymphoprep (Axis- Shield, Oslo, Norway). The PBMC were washed twice and centrifuged at 220 g for 8 min at 4°C, respectively, in order to further increase the leukocyte to platelet ratio. Monocytes were further isolated using the Monocyte Isolation Kit II (Miltenyi Biotec Norden AB, Lund, Sweden). In four experiments, additional anti-CD61 microbeads (Miltenyi Biotec Norden AB, Lund, Sweden) were added to reduce residual platelet numbers. The final untouched monocyte fraction was washed, counted on a CASY cell counter and resuspended in serum free CellGro DC medium (CellGenix GmbH, Freiburg, Germany). During culture, Nunclon Δ 6-well plates and Nunc HydroCell 6-well plates were used in comparison as representative standard culture dish and non-adherent culture dish, respectively (Thermo Fisher Scientific, Waltham, USA). For the MLR and the blocking antibody experiments, the non-adherent culture dish was changed to Nunc Sphera (Thermo Fisher Scientific, Waltham, USA), the newer line of non-adherent culture plates, due to discontinuation of the HydroCell series. 0.75 × 10^6^ monocytes/ml CellGro DC medium were plated per surface and per maturation stimulus, supplemented with IL-4 (20 ng/ml) and GM-CSF (100 ng/ml) (ImmunoTools, Friesoythe, Germany). IL-4 and GM-CSF were replenished after 2 days, 24 h before cell harvesting.

For 5 of the donors, blocking experiments were performed. For the other 14 donors, five different DC populations were generated for each surface, two of them immunogenic (LPS and Jonuleit cytokine cocktail, respectively), two tolerogenic (Dex/VD3 and IL-10, respectively) and one control sample without additional stimulus. LPS (100 ng/ml; Sigma-Aldrich, Taufkirchen, Germany) and the Jonuleit cytokine cocktail [10 ng/ml of IL-1β, 10 ng/ml of TNF, 1,000 U/ml of IL-6; all ImmunoTools, Friesoythe, Germany, and 1 μg/ml of prostaglandin E2 (PGE_2_); SigmaAldrich, Taufkirchen, Germany] were added 24 h before harvesting, respectively. For the generation of DexVD3 DC, 1 μM of dexamethasone (Dex; Sigma- Aldrich, Taufkirchen, Germany) was added at the start of culture, and replenished 24 h before harvesting together with 1 nM of 1α, 25-Dihydroxyvitamin D3 (VD3) (Enzo Life Sciences, Farmingdale, NY). For the generation of IL-10 DC, IL-10 (10 ng/ml) was added at culture start and replenished 24 h before harvesting (Miltenyi Biotec Norden AB, Lund, Sweden). As DMSO (Sigma-Aldrich, Taufkirchen, Germany) was used as a solvent for Dex and VD3, a corresponding amount of DMSO was added to the control samples (DMSO iDC). All cells were harvested after 3 days in culture. Cell-free supernatants were stored at −20°C for later cytokine detection. The remaining cells were washed off the surfaces with PBS (without magnesium and calcium; Lonza, Verviers, Belgium) containing 2 mM EDTA (Sigma- Aldrich, Taufkirchen, Germany). The viability of the generated DC population for each condition was determined by annexin-V and 7-AAD staining using the Annexin V Apoptosis Detection Kit (eFluor 450) from eBioscience (AH Diagnostics, Oslo, Norway) and a LSRFortessa cell analyzer (BD, Franklin Lakes, USA) located at the Core facility for Flow cytometry, Dept. of Clinical Science, University of Bergen.

### Blocking Antibodies

In some experiments, blocking antibodies against CD11a (clone HI111), CD11b (clone ICRF44), CD11c (clone 3.9), CD18 (clone TS1/18; all 10 μg/ml; BioLegend, San Diego, CA, USA), or E-cadherin (clone HECD-1; 10 μg/ml; Invitrogen/Thermo Fisher, Waltham, MA, USA), all low endotoxin, azide free, were added in the growth medium for 3 days during moDC generation. As a control, the moDC were cultured with mouse IgG1 (BioRad, Hercules, CA, USA) supplemented in the growth medium in the same concentrations as the blocking antibodies. A cell population with no additional antibodies served as a negative control. After 3 days of culture, the morphology of the generated moDC populations was analyzed by light microscopy using a Cytation 5 Cell imaging-reader (BioTek instruments, Winooski, VT, USA), before the cells were harvested for phenotyping.

### Immunostaining

The phenotype of the generated moDC populations was analyzed using the surface markers shown in Table 1. Cells were pre-incubated for 5 min using 2 μl of FcR blocking reagent (Miltenyi Biotec Norden AB, Lund, Sweden) per up to 10^6^ cells in 150 μl cold PBS containing 0.5 % bovine serum albumin (BSA; Sigma-Aldrich, Taufkirchen, Germany), followed by an incubation with the titrated amounts of antibodies for 15 min in the same buffer. The flow cytometry analysis was performed at the Core facility for Flow cytometry, Dept. of Clinical Science, University of Bergen, using a BD LSRFortessa cell analyzer.

**Table 1:** Mouse monoclonal anti-human antibodies with fluorophores and clone IDs used for flow cytometry analysis.

**Antigen**	**Fluorophore**	**Clone**
CD14	FITC	18D11
CD1a	PE	HI149
CD38	PerCP-Cy5.5	HIT2
CD83	PE-CF594	HB15e
CD40	PE-Cy7	5C3
CD86	Alexa Fluor 647	IT2.2
CD80	Brilliant violet 605	2D10
HLA-DR	Horizon V500	G46-6
CD197 (CCR7)	Brilliant violet 421	G043H7
CD18	FITC	TS1/18
CD11c	PerCP-Cy5.5	3.9
HLA-A,B,C	APC-Cy7	W6/32
CD274 (PD-L1)	PE-Cy7	MIH1
CD273 (PD-L2)	Alexa Fluor 647	MIH14
CD11b	Brilliant violet 421	ICRF44

### Cytokine Detection

For the detection of cytokines in the cell medium after DC generation, we used a magnetic microbead based 25-plex human cytokine kit for the Luminex platform (Invitrogen Corp., Carlsbad, USA). The cytokines measured were IL-1β, IL-10, IFN-α, IL-6, IL-12, RANTES (CCL5), Eotaxin (CCL11), IL-13, IL-15, IL-17, MIP-1α (CCL3), GM-CSF, MIP-1β (CCL4), MCP-1 (CCL2), IL-5, IFN-γ, TNF-α, IL1RA, IL-2, IL-7, IP-10 (CXCL10), IL-2R, MIG (CXCL9), IL-4, and IL-8. All supernatants were thawed and analyzed simultaneously. Measured median fluorescence intensity (MFI) values below the standard-curve were set to the detection limit and cytokines with MFI-values above the standard-curve were approximated by extrapolating linearly.

### Mixed Leukocyte Reaction (MLR)

In order to analyze the T cell stimulatory capacity of the generated moDC populations, we performed allogeneic mixed leukocyte reactions (MLR) as described previously (11). 5 × 10^4^ moDC were co-cultured with 2 × 10^5^ monocyte depleted PBMC stained with CFDA-SE (Vybrant CFDA-SE Cell Tracer Kit, Thermo Fisher Scientific, Waltham, USA) for 5–7 days in X-Vivo20 medium supplemented with IL-7 (10 ng/ml) and IL-2 (50 U/ml; both ImmunoTools, Friesoythe, Germany). At least 30,000 events were collected on a BD Accuri C6 instrument at the Core facility for Flow cytometry, University of Bergen.

### IFN-γ Secretion Assay

An IFN-γ secretion assay (Miltenyi Biotec, catalog number 130-054-202) was utilized to analyze the capacity of the generated DC populations to induce antigen specific T cell responses as described previously (11). In short, 2.5 × 10^6^ autologous monocyte-depleted PBMC were co-cultured with 5 × 10^5^ PPD-loaded DC populations for 7 days in X-Vivo20 medium supplemented with IL-7 (10 ng/ml) and IL-2 (50 U/ml). The IFN-γ secretion assay was performed according to the manufacturer's manual. As stimulators, PPD-loaded DC stimulated with LPS were used, and unloaded LPS-DC served as negative control. 2 × 10^5^ DC were co-cultured with 8 × 10^5^ induced monocyte-depleted PBMC for 16 h. Staphylococal enterotoxin B from Staphylococcus aureus (SEB; 1 μg/ml; Sigma-Aldrich) was added as positive control. Prior acquisition on a BD LSR Fortessa, the cells were stained with anti-CD4 FITC (M-T466, Miltenyi Biotec) and anti-CD8-APC (RPA-T8, Biolegend), as well as 7-AAD (ebioscience). A minimum of 2 × 10^5^ events were collected in the Lymphocyte gate. FlowJo was used to analyze the data, and % IFN producing cells were calculated according to the following formula:

$$\begin{array}{l} \%\text{ IFN}\gamma \text{ producing cells among CD}4^{+}=\\ \frac{\#\;of\text{ IFN}\gamma^{+}\text{ CD}4^{+}\text{ cells in the sample}}{\#\;of\text{ total CD}4^{+}\text{ cells in the sample}}\times 100 \end{array}$$

IFN-γ producing CD8+ T cells were calculated accordingly. The % IFN-γ producing cells from DC without PPD was subtracted from the values with PPD.

### Statistical Analysis

The statistical analyses were performed with Graph-Pad Prism (v5.02). Statistical significance was determined by comparing the cells of the two different surfaces for each treatment using a 2way ANOVA test in combination with the Bonferroni post-test or Dunn's post-test, significance criterion <0.05. Significance values in figures are given in grades *P* < 0.05 (*), *P* < 0.01 (^**^), and *P* < 0.001 (^***^). Median values for each surface and treatment are marked with a line. Measured values are given as the mean ± the standard deviation (SD) if not stated otherwise.

## Results

### The Lack of Surface Adherence Leads to Increased Homotypic Cluster Formation That Can Be Reduced by Blocking CD18

**Figure 1:**
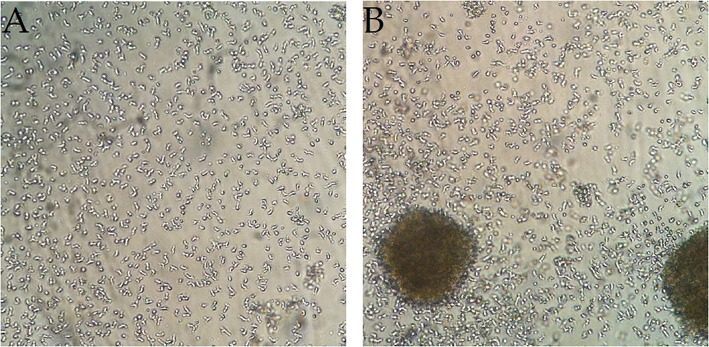
Homotypic cell clusters form on the non-adherent surface but less on the standard culture dish. Representative microscopy pictures of iDC (DMSO) at the end of the 3-day culture on **(A)** a standard cell culture dish and **(B)** a non-adherent culture dish. The increase in cell cluster formation on non-adherent dishes relative to standard dishes could be observed for all treatments (*n* = 8).

Using light microscopy, we observed an increase in cell cluster formation on the non-adherent culture dish in comparison to the standard cell culture dish for all moDC populations (Figure 1, Figure S1). Considerably smaller clusters were also observed on the standard cell culture dish, especially for the immunogenic stimulation conditions with LPS or the Jonuleit cocktail (Figure S1). Viability was not influenced by the different surfaces (data not shown). In order to determine the molecules responsible for this clustering behavior, we added various blocking antibodies to the culture. Blocking CD18 reduced clustering on the non-adherent plates (Figure S2), while blocking CD11a and CD11b appeared to promote homotypic clustering, independent of the surface used. Blocking CD11c and E-cadherin had inconsistent results.

### Culture Dish Adherence Influences Phenotype and Cytokine Production of moDC

**Figure 2:**
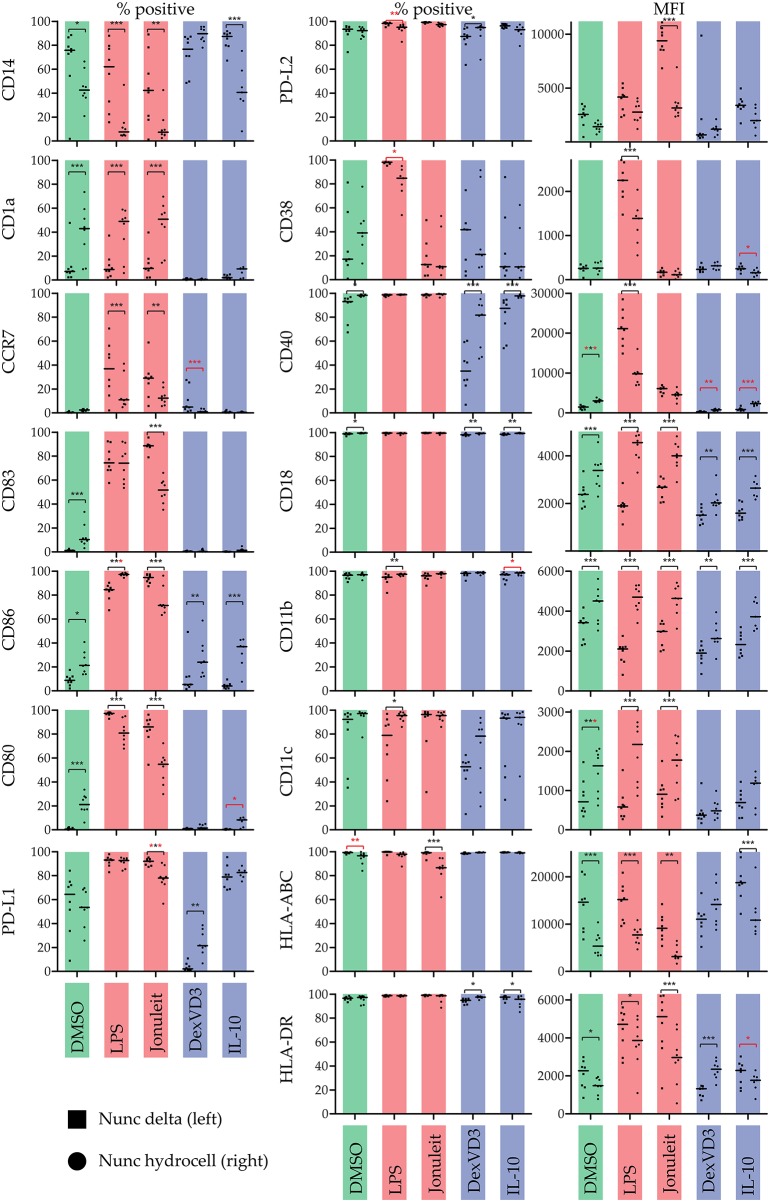
Influence of the culture dish on the phenotype of differentially stimulated moDC. The phenotype was analyzed by flow cytometry using the indicated surface molecules. % positive cells or median fluorescence intensity (MFI) are shown. Color code: Control (DMSO)—green, immunogenic DC populations (LPS & Jonuleit cytokine cocktail)—red, tolerogenic DC populations (Dexamethasone with vitamin D3 & IL-10)—blue. Squares **(left)**: standard culture dish; circles **(right)**: non-adherent culture dish. The median is marked with a line. Significance values are given in grades *P < 0.05, **P < 0.01, and ***P < 0.001. Black significance grades are the result of a collective 2 way ANOVA testing for all treatments, red grades are from separate tests for the iDC/tolDC and immunogen subgroups (*n* = 8).

We further investigated phenotypic differences depending on the treatment and surface conditions by analyzing expression levels of 15 different surface markers using flow cytometry (Figure 2). The expression of all cell surface molecules was influenced by the culture dish used. Interestingly, the use of a non-adherent culture dish decreased expression of PD-L1 on cells treated with the Jonuleit cocktail, while the other PD-ligand, PD-L2 (CD273), was highly expressed.

**Figure 3:**
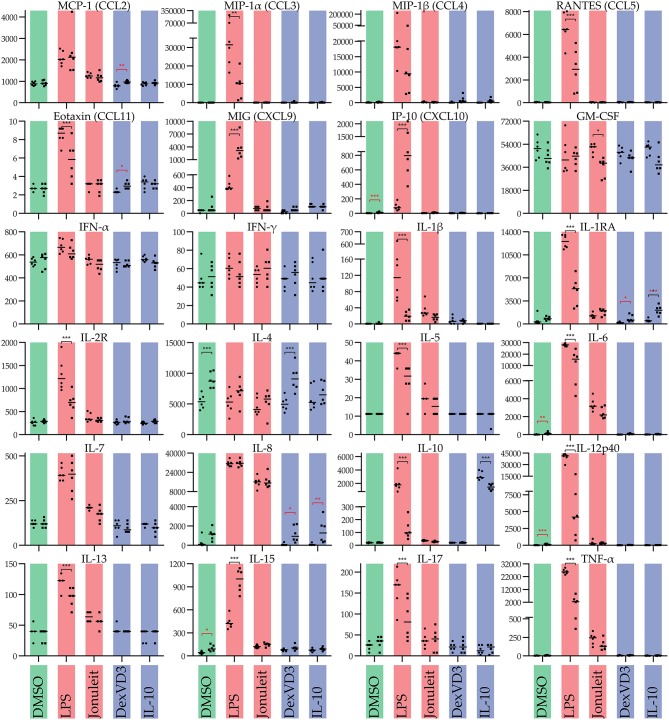
Influence of the culture dish on the cytokine production of differentially stimulated moDC. Cytokines were measured in cell free culture supernatants of the generated moDC populations. Color code: Control (DMSO)—green, immunogenic DC populations (LPS and Jonuleit cytokine cocktail, respectively)—red, tolerogenic DC populations (Dexamethasone with vitamin D3 and IL-10, respectively)—blue. Circles **(left)**: standard culture dish; squares **(right)**: non-adherent culture dish. The median is marked with a line. Significance values are given in grades *P < 0.05, **P < 0.01, and ***P < 0.001. Black significance grades are the result of a collective 2 way ANOVA testing for all treatments, red grades are from separate tests for the iDC/tolDC and immunogen subgroups (*n* = 6).

Cytokine production varied depending on the treatment and the surface used (Figure 3). In general, LPS-DC had the highest production of most cytokines. Regarding the influence of the culture dish surface, most cytokines were overall secreted at lower levels on non-adherent surface. Interestingly, the levels of exogenously added cytokines GM-CSF and IL-4 varied a lot. GM-CSF levels were higher on the standard cell culture dish than on the non-adherent surface, while IL-4 levels were lower. While the cytokine profile of the DC cultured in tolerogenic conditions as well as control showed limited differences in cytokine profile regardless of culture dish surface, the LPS stimulated DCs had significant profile differences. Interestingly, IL-10 and TNF-α secretion was increased in all LPS stimulated DCs on standard cell culture dish compared to non-adherent surface, while the opposite was true for IL-15 and MIG.

Blocking of adhesion molecules resulted in little differences in surface expression of most markers analyzed (data no shown).

### Additional Platelet Reduction During Monocyte Isolation Has No Distinct Effect on Phyenotype and Cytokine Production

In order to investigate the possibility that isolation impurities might have an impact on the clustering, phenotype, and cytokine production, four of the monocyte isolation procedures were performed using additional anti-CD61 beads to remove platelets. While the monocyte purity without the use of anti-CD61 beads was >85 %, it increased to >95 % when additional anti-CD61 beads were used (Figure S3), confirming that platelets were the main impurity. However, both the phenotype of the generated DC populations and their produced cytokines did not show any clear differences between the preparations with and without residual platelets (data not shown).

### The Cell Culture Surface Does Not Influence the T Cell Stimulatory Capacity of moDC

**Figure 4:**
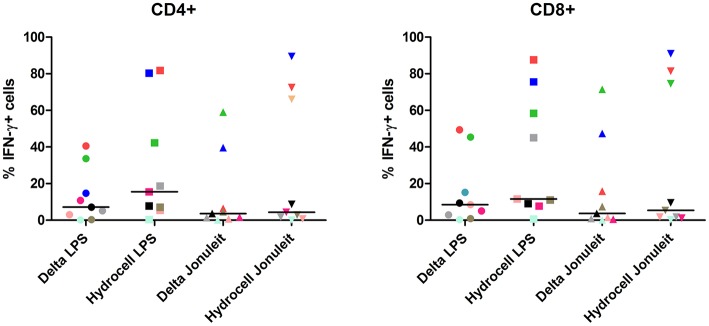
Antigen-specific T cell induction is not influenced by the culture dish conditions of the moDC. Autologous PBMC depleted of monocytes were co-cultured with PPD-loaded DC generated on the indicated surface and matured with indicated stimuli for 7 days, and antigen-specific IFN-γ secretion by CD4+ and CD8+ cells was analyzed by flow cytometry with LPS-DC as stimulators (± PPD). Delta LPS: moDC cultured on a conventional culture dish, stimulated with LPS; Hydrocell LPS: moDC cultured on a non-adherent culture dish, stimulated with LPS; Delta Jonuleit: moDC cultured on a conventional culture dish, stimulated with Jonuleit cytokine cocktail (TNF, IL-6, IL-1β, and PGE_2_); Hydrocell Jonuleit: moDC cultured on a non-adherent culture dish, stimulated with Jonuleit cytokine cocktail. Each color-coded symbol represents results from one individual (*n* = 9).

Lastly, we analyzed the T cell stimulatory capacity of the generated moDC populations. Using allogeneic MLR, no obvious differences were observed between the different surfaces, regardless of the DC population used as stimulator (Figure S4). As expected, the co-culture with the immunogenic DC populations (LPS and Jonuleit-cocktail stimulated cells) resulted in higher proliferation compared to the immature control DC. The proliferation of monocyte-depleted PBMC co-cultured with the tolerogenic moDC populations was even less than with immature DC. We further analyzed the immunogenic moDC populations in an autologous setting. Both LPS- and Jonuleit-cocktail stimulated cells were able to induce antigen specific autologous CD4+ and CD8+ T cells, with slightly higher numbers of IFN-γ producing T cells upon using LPS-stimulated DC, independent of the culture dish used (Figure 4).

## Discussion

In this study, we analyzed the influence of the cell culture surface on differentially stimulated moDC. The formation of cell clusters was increased for all cells cultured on the non-adherent culture dishes. Cell clusters on the standard culture dish were in comparison very small and only forming after immunogenic stimulation, suggesting a different clustering mechanism. The phenotype was in many cases significantly modulated depending on the cell culture dish. A total of 17 of the 25 measured cytokines were secreted at significantly different levels depending on the culture dish by moDC stimulated with LPS. However, the T cell stimulatory capacity was not influenced by the culture surface.

As immature DC are known to have tolerogenic functions (12), and based on the similarities in phenotype and cytokine production between the iDC and tolDC in our study, iDC/tolDC will be discussed collectively. In contrast, due to the distinct differences observed between LPS-DC and Jonuleit-DC, they will be discussed separately.

### Immature DC and Tolerogenic DC

The typical monocyte/macrophage marker CD14 was expressed at high levels in all iDC/tolDC populations on the standard culture dish, but at lower levels in the non-adherent culture dish, with the exception of DexVD3 DC. This is in line with a previous study showing that culturing monocytes in suspension rather than adherent conditions leads to a rapid reduction in CD14 (13). Thus, using CD14 as a marker for successful DC generation when comparing adherent with suspension protocols might not be the best choice. Nevertheless, the prevention of the suggested internalization of CD14 by DexVD3 treatment is interesting and should be investigated further.

The lack of DC maturation marker CD83 on all iDC/tolDC populations confirmed their immature state. Interestingly, iDC cultured on non-adherent surfaces showed a slight but statistically significant increase in CD1a, CD83, and CD80 expression, indicating that the formation of clusters might lead to a “spontaneous” DC maturation. This hypothesis is further strengthened by our observation that blocking CD11b, resulting in increased homotypic clustering on the non-adherent surface, also led to increased expression of costimulatory molecules and MHC. This effect was not observed in the other tolDC populations. However, further studies will have to investigate the effect of the clustering on the tolerogenic function of the different moDC populations *in vitro* and *in vivo*.

### LPS Matured DC

LPS-stimulated moDC on standard cell culture dishes were, as expected, CD83^+^ CD40^*high*^ CD86^*high*^ CD80^+^ MHC-II^*high*^ and increasingly CCR7^+^. Using a non-adherent culture dish leading to the formation of clusters had a great impact on the phenotype and cytokine secretion of the LPS-DC. Interestingly, based on the CD83 expression, the percentage of mature DC did not change, indicating that the observed changes reflect a change of polarity but not maturation. Especially interesting was the decrease in secretion of CC chemokine family members CCL3, CCL4, and CCL5, which are all ligands of CCR1 and CCR5. These chemokines attract mainly cells of the innate immune system like granulocytes (14), monocytes/macrophages (15), NK cells (16), mast cells (17), or immature dendritic cells (18) but also CD8^+^ T cells (16, 19). Interestingly, IL-8, another chemokine of the innate immune system attracting neutrophils (20), was not modulated and secreted in equally high amounts. Strikingly, the chemokines CXCL9 and CXCL10, both ERL-negative ligands of CXCR3, were significantly more secreted. Especially CXCL10 had been nearly absent on LPS-DC on the standard culture dish, but was secreted by moDC on the non-adherent culture dish. Both chemokines of the CXC family have been associated with supporting T helper 1 differentiation *in vivo* (21) but their receptor CXCR3 appears also to be essential during wound healing (22). CXCL9 and CXCL10 have also been reported to recruit activated IFN-γ expressing NK cells, CD8^+^ T cells and CD4^+^ T cells (23). The anti-inflammatory cytokines IL-1RA, IL-2R, and IL10 were also secreted at lower levels. Also IL-6 secretion was reduced considerably. IL-6 has been associated with maintaining immature DC (24) but is also linked to T helper 17 polarity (25). While IL-17 was not secreted as much, it also was reduced upon use of non-adherent culture dishes. Interestingly, IL-15 secretion was significantly increased by LPS-DC cultured on a non-adherent cell culture dish. IL-15 is a cytokine secreted mainly by monocytes/macrophages and dendritic cells (26, 27) and has gained a special interest as it is required for the differentiation of NK cells, effector CD8^+^ T cells and memory CD8^+^ T cells (26). IL-15 is also involved in antiviral immunity by formation of IL-15-IL-15Rα complexes able to induce IFN-γ mediated responses independent of type I IFN (23).

Taken together, LPS-DC shift from a CC chemokine response to a CXC chemokine response when modifying the culture dish from a standard cell culturing condition to a non-adherent cell culture dish. The high CD40 expression and high secretion of chemoattractants by cells cultured on the standard cell culture dish might be an example of an “all out” immune response, which is only kept under control by anti- inflammatory cytokines like IL-1RA, IL2R, and IL-10. The moderation of many of these factors on the non-adherent culture dish combined with an upregulation of CD86, IL-15, and the T cell chemoattractants CXCL9 and CXCL10 suggest a rather directed response, probably of a T helper 1 direction. Further *in vitro* and *in vivo* studies are needed to analyze the functional impact of the non-adherent culture dish during LPS- DC generation.

### DC Matured With Jonuleit Cytokine Cocktail

Interestingly, Jonuleit cytokine cocktail stimulated DC behaved very differently than LPS-DC. CD83 expression was significantly reduced when using a non-adherent cell culture dish, indicating less mature DC. Thus, most of the other observed changes in phenotype markers can be explained with the lower amount of mature DC. Surprisingly, there was no significant difference in the cytokine production except for GM-CSF between Jonuleit-DC cultured on standard cell culture and non-adherent cell culture dishes. In comparison to iDC/tolDC, most cytokines were not secreted significantly different.

Taken together, the impact of the change in cell culture surface seems more predictable for the Jonuleit DC. The use of a non-adherent culture dish will probably not induce a different polarity but would reduce the number of immunostimulatory DC. While this is interesting with regard to *in vivo* mechanisms controlling inflammation, a non-adherent cell culture surface might not be the best choice when aiming at high numbers of immunogenic DC to be used for immunotherapy, even though the T cell induction capacity was not impaired. However, we here used a recall antigen (PPD), and did not analyze the capacity to induce naive T cells, meaning the effect of culture dish adherence on DCs ability to process and present novel antigens remains to be explored. This suggests that future studies on the matter should consider utilizing another antigen with no prior memory presence and isolate naïve T-cells with negative selection prior to T cell induction capacity analysis. Another possibility to address this question would be to test the T cell induction capacity of DC on naïve T-cells from transgenic mice with known antigen specificity. In our experiments, the cytokines contained in the Jonuleit cocktail have rather broad inflammatory functions and give more of a “danger” stimulus, while LPS binds to TLR4, an innate pattern recognition receptor with specific function and predefined certainty of pathogenic recognition. The close contact with “self” in the clusters might rather calm down the unspecific stimulation. The LPS-DC on the other hand can rather be sure of an immediate danger and can than only be modulated to change polarity instead of remaining dormant. This gives an indication of when the change to the non-adherent culture conditions might lead to new subtypes of DC.

### Possible Mechanisms for the Induction of the Observed Culture Dish Dependent Differences

Based on microscopy observations, the main consequence of using a standard cell culture dish during moDC generation is an early adherence phase, while moDC cultured on a non-adherent surface form homotypic clusters. Usually, adherent monocytes will detach during the first day in DC culture conditions (10, 28). After the detachment, the characteristics of the cell culture dish should not influence the floating cells any longer. However, the early adhesion prevents the floating cells to cluster afterwards. It is unclear if the early adhesion is only important to control clustering or if the early adhesion alone already triggers signaling pathways which will lead to the phenotype differences, independent of the following clustering. Intriguingly, all three integrins analyzed, CD11b, CD11c, and CD18, were significantly higher expressed on moDC cultured on the non-adherent culture dishes (Figure 2). However, it is unclear if this is connected to the mechanism inducing the phenotype changes. Integrin-binding has been shown to induce DC maturation (29), and there is evidence suggesting that the conversion of monocytes to DC can be supported by specific integrin-binding (30).

Based on the high expression levels, we chose to utilize blocking antibodies against CD11a, CD11b, CD11c, CD18, and E-cadherin. It has been shown previously that CD11d/CD18 and CD11c/CD18 play a role in myeloid cell adhesion and spreading (31, 32). Surprisingly, only anti-CD18 consistently reduced the homotypic cluster formation in our study, suggesting other additional molecules to be involved. In contrast, blocking CD11b led to increased clustering in all our samples. This phenomenon has previously been observed by another study on blocking CD11b on moDC that suggests CD11b to be a competitive inhibitor of other more prominent integrins, thus resulting in stronger adhesive properties of moDC when blocked (33).

Homotypic clusters did not only form more intensively on the non-adherent surface, but they also persisted over days, thus showing a totally different dynamic as the early surface adhesion on the standard adherent dish. However, as detached monocytes on the standard dish did not form homotypic clusters prior to stimulation, there might also be a different integrin regulation involved in the homotypic aggregation at that point. Homotypic clustering or aggregation of DC has been observed *in vitro* and *in vivo*, but its natural function is unknown. However, support for both maturation and antigen-transfer as possible mechanism has been observed (34). It is tempting to speculate that differently matured cell types like infiltrating monocytes, locally developing DC and resident mature DC populations might cooperate in this way, helping immature cells to mature and transfer the original antigen-information to developing migrating cells in order to stimulate the adaptive immune system without having to abandon the site of inflammation. However, the effects reported and assigned to the homotypic cluster formation might also overlap with the reduction of integrin activation or other interactions of the cells with their surroundings. Further investigations will have to distinguish between these sources of influence. Future experiments should also address other culture surface conditions such as glass or other container conditions such as culture bags.

## Conclusions

The use of a non-adherent surface instead of a standard culture dish can have a great impact on the phenotype and the cytokine production of differently stimulated moDC. Further investigations will be required in order to elucidate the molecular mechanisms for the effect, but differences in the early direct surface interactions and in the frequency and amount of cell-cell interactions, influenced by homotypic cluster formation, might play a deciding role. This study proves that monocytes are crucially influenced by the near surrounding during the development into dendritic cells. This has a potential application for DC mediated immunotherapy, where the cellular phenotype is essential for the success of the treatment.

## Data Availability Statement

The datasets generated for this study are available on request to the corresponding author.

## Ethics Statement

The studies involving human participants were reviewed and approved by regional ethical committee for medical and health related research (#2009/686). The patients/participants provided their written informed consent to participate in this study.

## Author Contributions

AS and SA: conceived of the study. AS, SA, and DY: design of the study, analyzing data, and writing the manuscript. AS, DY, SR, and YL: generating data for the study. All authors approved the final manuscript.

### Conflict of Interest

The authors declare that the research was conducted in the absence of any commercial or financial relationships that could be construed as a potential conflict of interest.
