# Supplementary material for: The Culture Dish Surface Influences the Phenotype and Cytokine Production of Human Monocyte-Derived Dendritic Cells
Source: Front Immunol. 2019 Oct 2;10:2352. doi: 10.3389/fimmu.2019.02352 (PMC6783514; doi:10.3389/fimmu.2019.02352)
Supplement: Supplementary file 1 [file Image_1.pdf]

Supplementary file

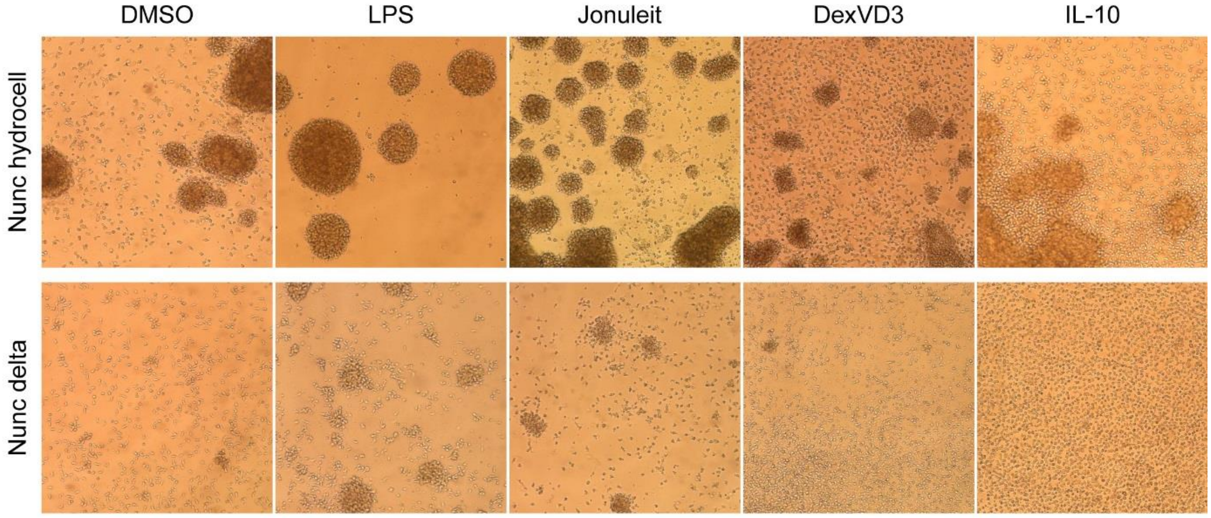

Figure S1: Homotypic cell clusters form on the non-adherent surface but less on the standard culture dish. Representative microscopy pictures of all generated DC populations at the end of the 3-day culture on a non-adherent culture dish (Nunc hydrocell) and a standard cell culture dish (Nunc delta). (n = 8)

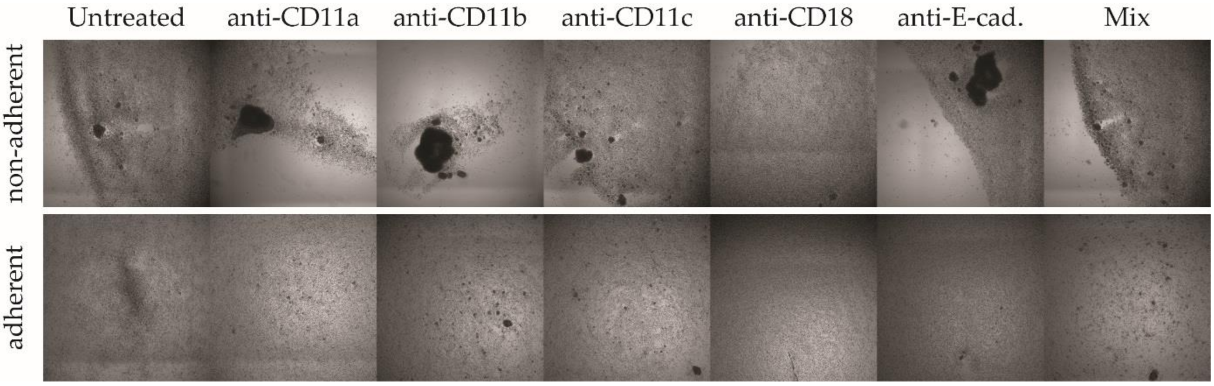

Figure S2: The effect of blocking cell adhesion molecules on homotypic cell clustering. Representative images of moDC cultured in non- adherent surface dish (upper row) and on standard surface dish (lower row) with the addition of IgG1 (untreated), anti-CD11a, anti-CD11b, anti-CD11c, anti-CD18, anti-E-Cadherin (anti-E-cad.) and a combination of all antibodies (Mix) after 3 days. Clustering on the non-adherent surface was notably reduced with the addition of anti-CD18 and increased with the addition of anti-CD11b. (n=5)

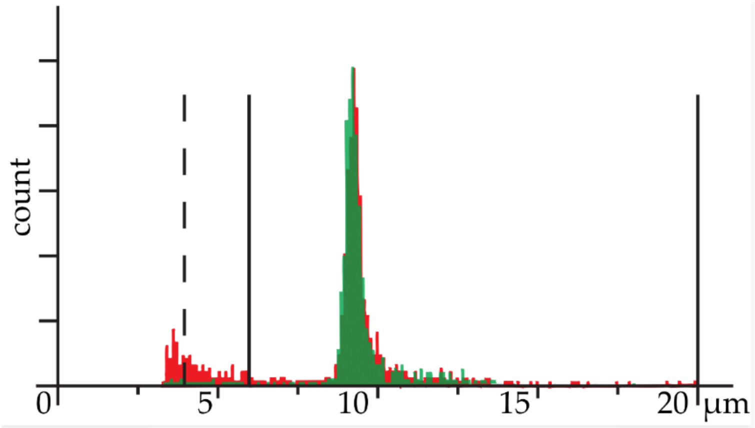

Figure S3: Improvement of the monocyte purity was achieved by further platelet removal. Representative Casy cell counter image overlay showing the initial monocyte purity with no further platelet removal (red) and with platelet removal using anti-CD61 microbeads (green). (n = 4)

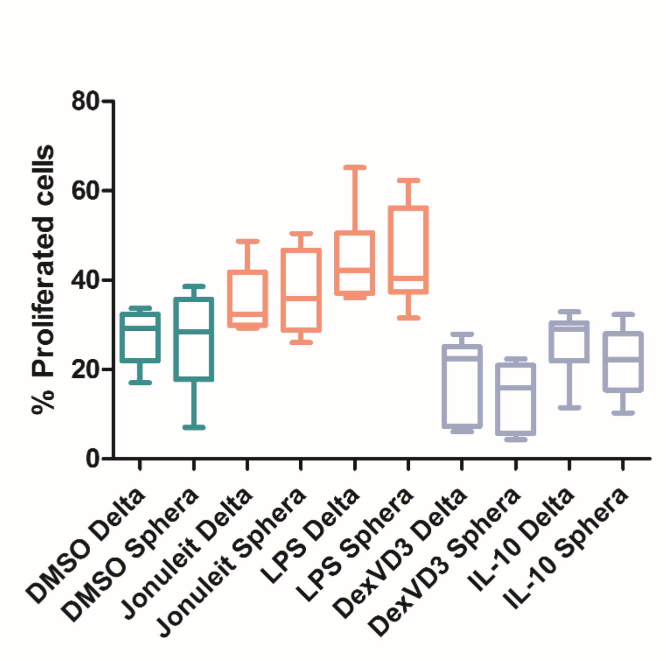

Figure S4: Mixed leukocyte reaction (MLR). Three-day moDC cultured on standard culture dish (Nunc Delta) or non-adherent culture dish (Nunc Sphera) with the addition of indicated compounds were subsequently co-cultured with allogeneic CFDA-SE stained monocyte depleted PBMC for 5-7 days. DMSO: immature moDC with DMSO control; Jonuleit: moDC stimulated with (TNF, IL-6, IL-1β and PGE2); LPS: moDC stimulated with LPS; DexVD3: moDC cultured with dexamethasone and VD3; IL-10: moDC cultured with IL-10. Percentage cell proliferation of monocyte depleted PBMC was analyzed by flow cytometry and shown as box plots with whiskers and median line. (n = 6)
